# Mapping the use of simulation in prehospital care – a literature review

**DOI:** 10.1186/1757-7241-22-22

**Published:** 2014-03-28

**Authors:** Anna Abelsson, Ingrid Rystedt, Björn-Ove Suserud, Lillemor Lindwall

**Affiliations:** 1 Department of Health Sciences, Karlstad University, Karlstad, Sweden; 2 School of Health Science, University of Borås, Borås, Sweden

**Keywords:** Simulation, Prehospital, Systematic literature review

## Abstract

**Background:**

High energy trauma is rare and, as a result, training of prehospital care providers often takes place during the real situation, with the patient as the object for the learning process. Such training could instead be carried out in the context of simulation, out of danger for both patients and personnel. The aim of this study was to provide an overview of the development and foci of research on simulation in prehospital care practice.

**Methods:**

An integrative literature review were used. Articles based on quantitative as well as qualitative research methods were included, resulting in a comprehensive overview of existing published research. For published articles to be included in the review, the focus of the article had to be prehospital care providers, in prehospital settings. Furthermore, included articles must target interventions that were carried out in a simulation context.

**Results:**

The volume of published research is distributed between 1984- 2012 and across the regions North America, Europe, Oceania, Asia and Middle East. The simulation methods used were manikins, films, images or paper, live actors, animals and virtual reality. The staff categories focused upon were paramedics, emergency medical technicians (EMTs), medical doctors (MDs), nurse and fire fighters. The main topics of published research on simulation with prehospital care providers included: Intubation, Trauma care, Cardiac Pulmonary Resuscitation (CPR), Ventilation and Triage.

**Conclusion:**

Simulation were described as a positive training and education method for prehospital medical staff. It provides opportunities to train assessment, treatment and implementation of procedures and devices under realistic conditions. It is crucial that the staff are familiar with and trained on the identified topics, i.e., intubation, trauma care, CPR, ventilation and triage, which all, to a very large degree, constitute prehospital care. Simulation plays an integral role in this. The current state of prehospital care, which this review reveals, includes inadequate skills of prehospital staff regarding ventilation and CPR, on both children and adults, the lack of skills in paediatric resuscitation and the lack of knowledge in assessing and managing burns victims. These circumstances suggest critical areas for further training and research, at both local and global levels.

## Introduction

Across the globe, prehospital settings are frequently associated with premature death [1]. With more rapid and more correct care efforts, some deaths due to high energy trauma could potentially be prevented [2]. However, training on accident scenes are hard to create, given that high energy trauma is rare. It is frequently also unsafe to use such unstable patients as training objects. Therefore, it is valuable to create real-life training opportunities in artificial contexts [3-5]. Simulation facilitates both the initial learning and repeated rehearsals of specific management of critical incidences [6]. It provides for training under optimal conditions [4], of importance in e.g. high-stake scenarios [7]. Regularly scheduled prehospital training opportunities would enable staff to be better prepared and more confident at trauma scenes.

Consequently, simulation plays an increasing role in prehospital care management training [6,8]. Therefore, research into its effectiveness and comparisons of instructional designs are increasingly important [4]. Research on simulation as a learning tool for prehospital care providers is still limited [3]. The field is a relatively young and still relatively unsystematised although some reviews have been published [4,9,10]. However, a more complete accounting of all existing studies is still lacking [9].

Consequently, the goal of this review is to create a map of the existing field of research on simulation in prehospital care settings. The overview can serve as a base for future research on methods, effectiveness, efficacy and/or technical skills.

The review documents historical developments, including developments in geographical regions. It is important to note that the presence of research on simulation in one region does not imply that prehospital care in this region necessarily is of better quality. However, in order to continue to develop learning opportunities and continual education, each region will likely benefit from research conducted in settings with reasonably similar culture, context, conditions and resources, as their own. Further, the review documents what methods were targeted in the simulation exercises and for whom, i.e., what professional categories were the foci of the simulation exercises, as research participants. Hopefully, this overview of research may suggest knowledge gaps and important next steps regarding simulation in the prehospital care settings. Furthermore, it may act as stimuli for research on simulation in prehospital contexts, across geographical areas and with methodologies which have not yet been prioritized.

The aim of this study was to provide an overview of the development and foci of research on simulation in prehospital care practice.

## Methods

An integrative literature review was carried out [11-13]. The integrative method allowed for the inclusion of studies from several different research methods. It plays a significant role in evidence-based practice as a more comprehensive understanding of specific problems within healthcare is formed [13].

### Search strategies

Electronic searches were conducted in the databases Cinahl, Pubmed and Scopus during the month of February, 2013. The searches were carefully documented [13]. Search terms which were used included: *emergency medical technicians, paramedic, manikin, simulation, ambulance* and *prehospital*. Peer reviewed journals were included in Cinahl, in order to get the same results in Scopus, the search was limited to articles. Further combinations of search terms were carried out with the words: *emergency, trauma, model, anatomic, training* and *education*. These new combinations, however, generated no new references. The search utilized the Internet as well as reference lists of existing articles.

### Selection

The inclusion criteria were articles published as primary research, quantitative as well as qualitative studies. Furthermore, the research participants in included articles should be prehospital care providers, including paramedics, EMTs, MDs, nurses and fire fighters performing or participating in some kind of simulation. The simulation should have been performed in a prehospital context. Finally, the articles should have been peer-reviewed and published before January 1^st^, 2013. If non-English articles were to be included, an abstract in English was required.

The electronic search, inspired by Jadad et al [14] and Oxman [15], generated 865 hits, of which 147 were duplicates, resulting in 718 articles to review. Two persons then independently read titles and abstracts in order to identify studies that matched the aim and the inclusion criteria of the review. A total of 243 relevant articles were downloaded or ordered as full-text versions. The 243 retrieved articles were subsequently read in full by one reviewer (AA) to confirm the relevance to the purpose of the review and to ensure that the inclusion criteria were met. Consequently, out of the 243 articles, 165 were included in the study. Of these 165 articles originated 111 from Cinahl, published between 1989 and 2012. Forty articles of the 165 articles were located in PubMed, published between 1989 and 2012. Finally, 14 articles of the 165 articles were identified in Scopus, published between 1984 and 2012 (Table 1).

**Table 1:** The selection process

**Database**	**Number of hits**	**Full article retrieved and reviewed**	**Included**
Cinahl	639	176	111
Pubmed	104 (49*) = 55	51	40
Scopus	122 (98*) = 24	16	14
Total number of studies	718	243	165

*Duplicates.

Three additional reviewers (IR, BOS and LL) read and assessed samples of the 165 articles, in order to confirm that they made the same conclusions regarding inclusion into the review. This contributed to making the selection process transparent and thorough, as suggested by Oxman [15]. The reviewers reached a high level of agreement in the assessment process and any differences were discussed and agreement reached. Jadad et al [14] argue that it is ideal that all relevant literature on the subject is included in a review. Given that the purpose of the review was to provide an overview of research, it was therefore decided to include all 165 studies in the results.

### Data analysis

A systematic data analysis was conducted in accordance with Whittermore and Knafl [13]. The material was grouped in accordance to findings that answered the aim and formed into groups: history, geography, staff and five topics of simulation research. Qualitative and quantitative findings complemented each other given that the research addressed different but yet connected questions. The approach also enabled us to combine data from different studies and a scope could be assembled to create an overview over existing research [11-13].

## Results

The results illustrate how the volume of published research has been distributed through time as well as across regions. It similarly reveals what simulation methods and what staff categories are generally the focus of research. Finally, as a consequence of the aim of mapping the extent of existing studies, this review reflects details on the prehospital topics most extensively covered in the field: *Intubation, Trauma care, CPR, Ventilation and Triage.*

### History and geography

The aim was to provide an overview of development and foci of research on simulation in prehospital practice. Only a limited number of countries, worldwide, have published research on simulation with prehospital medical staff. The number of published articles increased markedly around the year of 2000. In the review, 85 published articles originate from the US. At the turn of the millennium, similar research started to be carried out and published in UK and Canada, and a few years later in other countries. UK was the first European country to regularly publish research on simulation in the context of prehospital care, several years of Germany and Scandinavia, (Table 2).

**Table 2:** Published articles distributed by year and continent

	**1984-1988**	**1989-1993**	**1994-1998**	**1999-2003**	**2004-2008**	**2009-2013**	**Total**
North America	1	6	10	12	37	29	95
Europe		1	6	1	7	34	49
Oceania				1	4	5	10
Asia				2	1	4	7
Middle East				1		3	4

Through 2010, 55% of published articles have originated from North America. During the same time frame, European published articles represent 30% of the total, and approximately 5% have originated from Oceania and Asia. After 2010, in comparison to before 2010, the share of published articles on simulation in the prehospital context from Europe has increased, whereas the North American share has decreased.

### Simulation methods focused on

The published research focused on a number of simulation methods. Simulation with manikins was the most common method studied across all regions (143 studies). Simulations with films, images or paper as tools were the focus on research in 14 of the published studies. In Asia; films, images and paper represented approximately 40% of their methods evaluated. Compared to only 5% of the published articles in North America. Live actors, as a simulation tool, were used in 12 studies (11 were from North America), whereas cadaver were used in 6 studies (5 from North America). Finally, virtual reality as the method of simulation of prehospital care situations was used in 3 studies (all from North America). Some studies integrated several simulation methods in the same published article and were included in all the different methods.

### Research participants in simulation exercises

Overall, published research on simulation in the context of prehospital care (n = 165) was, in the review, conducted with the following professions as participants: paramedics (n = 111, 46%), EMTs (n = 59, 24%), MDs (n = 34, 14%), nurses (n = 31, 13%) and fire-fighters (n = 7, 3%). More than half of the research on simulation published across all regions focused on paramedics. During the past five years, research on simulation focusing on paramedics has increased while, concurrently, the share of published research articles focusing on EMTs has declined. The share of published studies focusing on MDs and on nurses remains stable.

### Topics focused on in simulation

The specific foci of the simulation exercises which were reported on the published articles revolved around five areas of methods for and/or techniques for prehospital care: *Intubation, Trauma care, CPR, Ventilation* and *Triage.*

### Intubation

Intubation was the primary focus of research on simulation (36% of the articles). Research on simulation exercises focusing on intubation started in 1996, and intubation has since then continued to be a common focus of simulation research (Table 3).

**Table 3:** Number of articles focusing on intubation, published by continent 1984-2012

	
North America	38
Europe	15
Oceania	3
Middle East	2
Asia	1
Total	59

Most of the published articles on simulation and intubation were carried out with paramedics (58%) as research participants, only one article on intubation involved military staff. Among the published simulation studies which targeted intubation, comparisons of different laryngoscopes or tubes was the most common (70%), including studies on different techniques for intubation/Bougie assisted intubation/free airway [16-52].

The remaining articles (30%) highlighted the positive effects on learning and improved skills as a consequence of intubation training [53-66]. Some articles described how the intubation was affected by positioning of staff and patient, as well as by external conditions [67-76].

### Trauma care

Trauma care, including prehospital procedures, was the focus of 32% of published research on simulation exercises. Most research on simulation with trauma care as the specific focus has been published after 2000 (Table 4).

**Table 4:** Number of articles focusing on trauma care, published by continent 1984-2012

	
North America	33
Europe	12
Oceania	6
Middle East	2
Asia	0
Total	53

Research participants in simulations focusing on trauma care included paramedics (44% of all articles), EMT’s (22%) and MDs (20%). Seven articles on simulation in the context of trauma care targeted military staff.

Simulation exercises focusing on trauma care basically concerned simulation training methodology and staff performance, which included paediatric resuscitation, intraosseous needle insertion and intranasal medication. They also targeted the assessment of, for example, burn victims, blood loss or immobilisation of the trauma patient. A smaller share described the prehospital work environment.

#### Simulation as a method for education in trauma care

Several of the studies focused on how fidelity as well as simulation method, in themselves, impacted the experience of the simulation exercise [77-91]. These articles highlighted that simulation provided a positive and helpful opportunity to train under stress [92-95] and was an effective way to train for biological, chemical, nuclear and terror attack scenarios [96-100].

#### Performance

A research study focusing on paediatric resuscitation skills concluded that the performance of the paramedics were deficient in one way or another regarding infant cardiopulmonary arrest (55% of the paramedics did not perform this correctly), infant respiratory arrest (48%) and infant sepsis (53%) [101] which suggest a need for continuous staff education in paediatric trauma care.

Three studies regarding intraosseous needle insertion supported that it was easy to learn for the prehospital provider with a satisfactory success rate and few complications [102-104] as was administering intranasal medication when performed by 18 advanced paramedic trainees in a randomised controlled trial [105]. This indicated that both intraosseous needle insertion and intranasal medication is something that could be used more frequently in the prehospital care. A similar circumstance was observed in the context of ultrasound were 2 studies showed that correct use of ultrasound was achieved by the prehospital staff after relatively little training [106,107] which indicated that ultrasound could be used diagnostically in prehospital situations. Studies showed that certain types of performance could be improved with external help. If the staff had access to physicians via telemedicine a more advanced care could be ensured, including caring for major trauma and myocardial infarction successfully performing needle thoracostomy and pericardiocentesis [108,109].

Limitations occurred regarding staff’s clothing where the study showed that working with protective clothing prolonged the prehospital care-taking process [110,111], as did working with night goggles in a randomized clinical trial when performed by 26 emergency physicians and paramedics [112]. At certain prehospital situations, the patient’s clothing instead posed a limitation because of the risk of hyperthermia. Two studies focused on desired and un-desired cooling of the patient. One study showed that removing wet clothing and using windproof and compression resistant outer ensemble protected against cold [113,114] while regularly changing cooled intravenous infusions resulted in desired patient hypothermia [115].

It also appeared that there were some limitation in staff’s willingness to restrain an agitated or violent patient, even though the staff had training in restraining [116] as well as the functionality of various types of tourniquet when performed by 10 military EMTs [117].

#### Assessment

Ten articles focusing on simulation of trauma care scenarios aimed at assessment. Two articles emphasised that there was a lack of training on the management of burns victims [118,119]. One of this studies, performed with 198 participants, showed that prehospital staff frequently underestimated the total burned surface area burned of the burn victims. This resulted in only 13% of the calculations of resuscitation fluid being correct (118). The other was a comparative simulation study, which showed that 125 emergency service and military paramedic staff over or under estimated the burn area to approximately 50% of 10 burn victims (119). Both these results indicate that the survival outcome diminishes.

When it came to immobilisation of trauma patients, training and experience resulted in 10 paramedics and 10 MDs made the same assessment in a randomized, prospective study. This demonstrated that the paramedics were able to reliably evaluate patients for possible cervical spinal injury [120]. But regardless of training or experience, the assessment of the patient’s loss of blood on the ground was in 3 studies regarded as too inaccurate to be of significance in the actual care [121-123], which point to the prehospital staff not taking time on the scene of the accident to assess blood losses.

#### Work environment

Two studies focused on work environment and showed that carrying stretchers and being exposed to prehospital working postures burdened the body [124-126], while vibrations in the cabin of a controlled ambulance transport could be reduced by using mattresses [127].

### Cardiac pulmonary resuscitation (CPR)

CPR represented 20% of the published research on simulation. Seven articles were published on CPR between 1995 and 2005, and since then, 2-4 articles have been published on CPR annually, resulting in a total of 35 articles (Table 5). Research on CPR declined somewhat between the years 2008 and 2012.

**Table 5:** Number of articles focusing on CPR, published by continent 1984-2012

	
North America	11
Europe	19
Oceania	0
Middle East	0
Asia	5
Total	35

The majority of the published articles, 43%, focused on how EMT: s carried out CPR, whereas 33% focused on paramedics and 8% on fire fighters. Only one article that focused on military staff was found.

Poor CPR implementation during simulation was identified in 8 studies [128-135]. According to 6 studies the participants performed inadequate compressions in 32% - 62% of the times [129-135] and had misplaced hands in 36% - 55% [129-131]. It was also found in two studies that 50% - 90% of ventilations during CPR were incorrectly performed [129,130]. The quality of CPR was relatively similar regardless of group of staff. Participants in 3 studies were not able to accomplish CPR that would likely have been of clinical benefit for an actual patient. The number of people performing CPR affected the implementation [136-138], as did the positioning of the staff [134,139-141]. CPR during simulation was also affected by external factors. CPR was described as possible to perform with adequate quality during surf lifesaving [142] but was negatively affected by such factors as car movement or carrying of stretcher [133,135,143,144]. The compression rate while performing CPR, varied also due to external factors such as audible feedback [131] which resulted in improved compression competence over time when training with a voice-assisted manikin [145]. External factors, such as dressing in protective clothing, prolonged the time taken for implementation of CPR [146,147] while the removal of a patient’s clothes using a cutter affected positively as it was quicker than using scissors in a manikin study when performed by 10 persons [148].

Articles differed regarding the use of external mechanical compressions instead of compressions performed by the prehospital staff. Both positive and negative effects were identified [149-155]. Malposition of the mechanical compression device counteracted the benefit of mechanical chest compressions. Still, participants using the external mechanical compression device adhered more closely to CPR guidelines than participants using active compression-decompression (ACD) CPR. ACD CPR caused a reduction of compression quality and many participants regarded ACD CPR difficult to use due to not being tall enough to apply the device [149-152]. Hands off time, i.e. time without compressions, was described as longer when using a mechanical device than manual compressions [153]. When using feedback devices regarding chest compressions these were described to overestimate the depth but withhold the compression efficacy [154,155].

The use of automatic defibrillation was described as quicker than manual defibrillation in the field, in a randomized simulation study which included 74 military medical participants [156]. Furthermore, reduced hands-off time was the result of manual defibrillation [157]. Both high and low fidelity simulation of cardiac arrest resulted in satisfied participants [158].

### Ventilation

Between the years 1989 and 1994, 6 articles were published on simulation in the context of ventilation, representing 6% of the total research articles. Following 1994, simulation regarding ventilation disappeared as an area of research and never quite returned. Only 5 articles focusing on simulation and ventilation were published in the years 2005-2011. Between 2008 and 2012 publications followed the same trend and stayed approximately the same as earlier, constituting 5% of the research with a total of 11 articles published (Table 6).

**Table 6:** Number of articles focusing on ventilation, published by continent 1984-2012

	
North America	7
Europe	2
Oceania	1
Middle East	0
Asia	1
Total	11

Research on simulation and ventilation has mainly been carried out with paramedics as the research participants, 50% of all articles focusing on ventilation and with EMTs, 29%.

The articles focusing on ventilation emphasised that ventilation was difficult and often had an unsatisfying result. Accordingly to two studies, to improve the quality of ventilation during CPR, a higher number of staff involved in the patient care was needed [159]. In addition, the ventilation quality was improved when the ventilation was performed mouth-to-mouth. However given that prehospital care providers may consider mouth-to-mouth ventilation unacceptable for regular use, a pocket mask with oxygen inlet was suggested [160], even though ventilation using a pocket mask was described as difficult. Three studies showed that adequate ventilation with mouth to mask on children was performed in between only 40- 75%, depending on the type of pocket mask devices being used [161,162] and with 20% of excessive pressure breaths [163].

Also bag- valve- mask showed, in 3 studies, difficult for the participants to use. When conducting bag- valve- mask ventilation, 60 out of 70 participants did not achieve adequate tidal volume in a paediatric manikin. Bag- mask- ventilation was associated with a significant large percentage, 56%, of excessive pressure breaths, [160,163,164]. One study showed that smaller 1-litre bags yielded better results than bigger 1,6 litre bags when performed by 30 paramedic students [165]. Also, a mechanical bag controller was shown to provide a small advantage compared to manual bag ventilation [166]. On the contrary, demand valve and automatic ventilators did not provide satisfactory ventilation on non-intubated patients in a comparison study performed by 15 EMTs [167].

Staff auscultation skills on intubated patients could be improved by training with simulated heart and lung sounds [168]. However, the only quick way of discovering a dislocated tube was to use capnography [169].

### Triage

Triage targeted 6% of the published research articles on simulation. A total of 10 articles on simulation and triage have been published since 2001; 6 in North America, 2 in Europe and Asia, respectively. The research underlying the articles focused on triage carried out with paramedics as research participants, 31%, whereas 25%, respectively, has been carried out with a focus on EMTs and MDs as research participants.

The articles focused on triage demonstrated that triage training improved the staff’s knowledge [170-173] including virtual reality training performed by 182 nurses, physicians and paramedics [174]. Table top exercise when performed by 59 EMTs was not evaluated as ideal due to the lack of actual implementation with equipment [175]. But if floor top exercise were performed with addition film screening of previous disaster drills it was experienced to provide the participants with a clearer picture of the situation [176]. If live victims scenarios were used instead, the participants scored this higher than written scenarios without significant differences and were, by 61 prehospital providers in an exploratory study, compared favourably, to manikins. This suggests that the two methods, live victims and written scenarios, although different in regards to cost, time- and space consumption may result in similar learning outcomes [177].

Using decision-making material resulted, according to a comparison study including 93 emergency service personal, in a more correct and quicker assessment [178]. It also improved participant confidence in a prospect observational study conducted with 73 in- and prehospital participants [179]. This suggests that some sort of support material works favourable for prehospital triage decision-making.

## Discussion

This study suggests there are relatively few published articles focusing on simulation in prehospital healthcare. The number of articles has increased in the last few years with a significant number of articles originating from North America. The research of a few countries is currently being applied in the whole world while cultural differences may imply that some countries would benefit from more local research on simulation in prehospital care. As the prehospital conditions and educations vary across different continents, there may be a need for research on simulation with other professions of prehospital care providers. The most frequently occurring research participants in this review were paramedics. However, given that EMTs are also common staff category in prehospital healthcare, simulation research with a specific focus on EMTs may need to increase. What is completely lacking in the result is the staffs’ relation to the patient. This may be disregarded because there are no patients involved rather different types of simulation. Nevertheless, it might be of interest to elevate the patient in this context as all care-giving is for the patient.

The area which was the focus of most published articles on simulation was intubation, while ventilation was most infrequently the focus. Ventilation is a necessity for patient survival and it is important that, regardless of scenario or injury, the staff is able to manage to uphold free air passage and to ensure the supply of oxygen. World Health Organization (WHO) similarly highlights the importance of training in assisted ventilation using different types of ventilation devices (pocket mask, bag-valve-mask etc.) for both adults and infants [1]. But the knowledge on behalf of staff to ventilate adults and children was across the studies described as inadequate. This resulted for example in a performance deficiency of 25- 86% for infant ventilation. Also the lack of skills in paediatric resuscitation was revealed in our study. Infants were seldom encountered in the studies which resulted in a gap of knowledge. This indicates a strong need for education and training in the prehospital care of infants.

Furthermore, survival outcome for burns victims diminished due to a lack of knowledge in estimating burned surface area. Burns victims were also seldom encountered in the studies and the staff had therefor inadequate knowledge in assessment and treating burn victims. WHO highlight the need to be able to assess degree of burns in context of depth and extent and the treat the burn victim, which they mean is essential for, advanced prehospital care providers to know [1]. Therefore, a shift of simulation research focus towards also integrating ventilation, paediatric resuscitation and management of burns victims may be beneficial. Reliable and easily accomplished techniques focusing on infants as well as burns could preferably be taught by means of simulation techniques. Subsequently, these techniques need to be followed-up and maintained to ensure adequate quality of care and patient safety. Simulation has a vital role to play in this follow-up.

With regards to CPR, in our review, poor CPR implementation during simulation identified staff not being able to accomplish CPR that would have been of clinical benefit for the actual patient. Ventilation and compressions had high percentage of incorrect performance on both adults and children. When giving CPR, it was revealed that 50% - 90% of the ventilations, and 32-62% of the compressions were incorrectly performed. Bobrow [180] points to the gap between the perceived performance of CPR and the CPR that is actually performed. Already in 1999, Liberman emphasised the benefit and importance of staff receiving feedback on how they perform CPR [129]. Such verification, with a range of simulation scenarios, could be repeated with varying degrees of difficulty to accommodate each participant’s pace of learning [4,5]. Simulation is important in building, improving and maintaining necessary skills among prehospital care providers [181], and there is an existing confirmed need for this [182]. However, Sanddal [172] notes that research needs to examine whether staged scenarios indeed are comparable to real events, something that lacks in evidence today. Future research will be particularly meaningful if the shortcomings that have been discovered by means of simulation are followed up with studies targeting on training and interventions. There is a lack of research within this area today.

The present review has highlighted published research within prehospital simulation research, an overview that might be valuable for decision regarding the direction of future research. It is important to continually build well-founded research based knowledge about how simulation can be utilised to further staff skills and knowledge, in order to better manage the relatively rare situations when high energy trauma occurs in real-life. Future papers are needed and can do more justice to the sub-areas discovered in this overall review.

### Limitations

The search was limited to years 1984- 2013. No countries or continents were excluded. The research participants were divided in groups where paramedics and nurses represent separate groups, given that this study adhered to description of the staff in each individual article. One limitation is that studies not indexed with the word simulation were not included. This means that studies that may have been of interest were not identified. Another limitation is that the included studies were not conducted on homogenous conditions as different cultures have varying conditions for prehospital healthcare and healthcare education. Yet another limitation is that the included articles were not subjected to a quality review, due to the primary purpose being to map the overall the research area. Whether quality should be assessed or not is, according to Whittermore and Knafl [13], controversial, as quality can often be confirmed but it is difficult to define or measure. Quality is assessed according to the information presented in the studies, which can incorrectly be interpreted as being of high or low quality depending on the manner in which it is reported [14]. In order to ensure validity in the present study, the method has been carefully described in order to enable reproduction of this study. Further, only peer-reviewed articles have been used [15].

## Conclusion

Simulation is described as a positive training and education method for prehospital medical staff. It provides opportunities to train assessment, treatment and implementation of procedures and devices under realistic conditions. It is crucial that the staff has familiarity with and is well-trained for techniques in; intubation, trauma care, CPR, ventilation and triage, which all, to a very large degree, constitute prehospital care. Simulation plays an integral role in ensuring these skills and this level of comfort.

Previous research, as well as this review, indicates that the current state of prehospital care is inadequate in many aspects, e.g. insufficient skills for ventilation and CPR, on both children and adults, as well as paediatric resuscitation and assessment and care management of burns victims. This suggests critical areas where future simulation training and simulation research may be directed, both on a local and global level.

## Competing interests

The authors declare that they have no competing interests.

## Authors’ contributions

AA conducted the literature search, analysed the material and wrote the manuscript. IR, BOS and LL supervised the literature search, were the additional assessors and supervised the analysis of the material and the writing of the manuscript. All authors read and approved the final manuscript.
